# Cutting edges and therapeutic opportunities on tumor-associated macrophages in lung cancer

**DOI:** 10.3389/fimmu.2022.1007812

**Published:** 2022-11-02

**Authors:** Qin Hu, Gujie Wu, Runtian Wang, Huiyun Ma, Zhouwei Zhang, Qun Xue

**Affiliations:** ^1^ Research Center of Clinical Medicine, Affiliated Hospital of Nantong University, Nantong, China; ^2^ Medical School of Nantong University, Nantong University, Nantong, China; ^3^ Department of Oncology, The First Affiliated Hospital of Nanjing Medical University, Nanjing, China; ^4^ Department of Cardiothoracic Surgery, Affiliated Hospital of Nantong University, Nantong, China

**Keywords:** lung cancer, tumor microenvironment (TME), macrophages, anti-tumoral immunity, macrophage polarization

## Abstract

Lung cancer is a disease with remarkable heterogeneity. A deep understanding of the tumor microenvironment (TME) offers potential therapeutic strategies against this malignant disease. More and more attention has been paid to the roles of macrophages in the TME. This article briefly summarizes the origin of macrophages, the mutual regulation between anti-tumoral immunity and pro-tumoral statuses derived from macrophage polarization, and the therapeutic opportunities targeting alternately activated macrophages (AAM)-type macrophage polarization. Among them, cellular components including T cells, as well as acellular components represented by IL-4 and IL-13 are key regulators driving the polarization of AAM macrophages. Novel treatments targeting macrophage-associated mechanisms are mainly divided into small molecule inhibitors, monoclonal antibodies, and other therapies to re-acclimate AMM macrophages. Finally, we paid special attention to an immunosuppressive subgroup of macrophages with T cell immunoglobulin and mucin domain-3 (TIM-3) expression. Based on cellular interactions with cancer cells, TIM3+ macrophages facilitate the proliferation and progression of cancer cells, yet this process exposes targets blocking the ligand-receptor recognition. To sum up, this is a systematic review on the mechanism of tumor-associated macrophages (TAM) polarization, therapeutic strategies and the biological functions of Tim-3 positive macrophages that aims to provide new insights into the pathogenesis and treatment of lung cancer.

## Introduction

Lung cancer is the second most common cancer worldwide and the leading cause of death (1). In recent years, the morbidity and mortality of lung cancer have accelerated significantly. Taking the United States as an example, it is estimated that there will be 1,898,160 new cancer cases in 2021, of which lung cancer ranks second in both male and female patients, accounting for 12% and 13%, respectively. And among the 608,570 estimated cancer deaths, lung cancer ranks first in mortality. Lung cancer is the leading cause of cancer death among men in both developed and underdeveloped countries (2). According to the clinical histological characteristics, lung cancer is mainly divided into small cell lung cancer (SCLC) and non-small cell lung cancer (NSCLC) which make up 85% of all cases. The common subtypes of NSCLC mainly include lung adenocarcinoma, lung squamous cell carcinoma (LUSC) and large cell carcinoma (3, 4). The annual survival rate of lung cancer cases is only 15.9%, and this data has only improved slightly over the past few decades (5).

The TME is the environment surrounding tumor cells. The TME is heterogeneous and consists of immune cells, fibroblasts, endothelial cells and neuronal cells, their extracellular matrix (ECM) proteins, signaling molecules and surrounding blood vessels (6). The TME is closely related with tumorigenesis and cancer progression through multiple mechanisms, including promoting epithelial-to-mesenchymal transition (EMT), facilitating tumor infiltration and contributing to immune suppression (7). The lung cancer microenvironment is characterized with prominent intra-tumoral heterogeneity, which could be caused by the heterogeneity of TME including mechanical properties, acidity conditions, and signaling molecules (8). A full understanding of the TME will facilitate the further development of effective therapies for lung cancer. In this review, we focused on TAMs, a critical component of the TME that plays an important role in the pathogenesis of lung cancer. We discussed the mutual regulation between anti-tumoral immunity and pro-tumoral statuses derived from macrophage polarization, and explore potential therapeutic opportunities targeting alternately activated macrophages (AAM)-type macrophage polarization in lung cancer.

## Macrophages: An important component in the immune microenvironment of lung cancer

Tumors are increasingly seen as complex ‘ecosystems’ where multiple interactions take place among cancer cells, immune cells as well as various components in the extracellular matrix (ECM) (9). The ECM comprises the majority of non-cellular TME, such as laminin, collagen, and fibronectin, while the cellular components surrounding tumor cells include immune cells (such as lymphocytes, NK cells, macrophages and dendritic cells) and non-immune cells (such as fibroblasts and vascular endothelial cells), collectively determining their roles in tumorigenesis and tumor progression. More and more evidence suggested that instead of driving uncontrollable proliferation and distant metastases on its own, cancer cells interact with the TME cells to re-shape the lesion into an immunosuppressive, chronic inflammatory, and pro-angiogenic microenvironment (10, 11). During the early stage of tumorigenesis, TME cells including the infiltrating inflammatory cells, endothelial progenitor cells, and cancer-associated fibroblasts constituted the infrastructure of cancer niches. With the proliferation of cancer cells, more immune cells infiltrated in. According to its role in carcinogenesis, TME cells could be divided into pro- and anti-tumoral components (12). Anti-tumoral macrophages, lymphocytes, natural killer (NK) cells, and dendritic cells (DC), which originated from the host microenvironment or recruited from the circulating system, were inhibited and acclimated by the immunosuppressive components, represented by myeloid-derived suppresser cells (MDSC), regulatory T (Treg) cells and M2 subtype macrophages (also known as tumor-associated macrophages, TAM) (10). M2-polarized macrophages can secrete interleukins that promote lung cancer tumorigenesis and metastasis. In turn, some interleukins can prime macrophage M2-polarization through stimulating the expression of interleukin receptors (13). Initially, macrophages performed both phagocytosis and antigen-presentation, while TAMs nourished tumor cells through a multitude of signaling pathways, hindered effector cells from attacking cancer cells, and promoted the occurrence, development and metastasis of malignant cancers (14–17).

### Origin of macrophages

In view of its tissue of origin, macrophages can be divided into two main subtypes. Belong to the mononuclear phagocytic system (MPS), some macrophages are differentiated from monocytes that were released from the bone marrow (18). Other tissue macrophages were derived from embryonic progenitor cells, maintained by *in situ* self-renewal without being replenished from the bone marrow (19–21). For example, in the epidermis and central nervous system, the vast majority of macrophages were maintained in a self-renewing manner instead from the recruitment of circulating monocytes (19, 22, 23). However, in the spleen and gut, bone marrow-derived macrophages contributed more (20, 24). Additionally, based on pathways of activation, macrophages were classified into “activated” macrophages involved in Th1-response and “alternatively activated” macrophages involved in Th2-response, and some researchers proposed that antigen-presenting DC in the circulation were also a member of the MPS lineage (14). Under pathological conditions, the monocyte/macrophage distribution was re-arranged. For instance, cytomegalovirus infection resulted in an accumulation of MPS cells in the bone marrow whereas a decrease in the peripheral circulation (17). Another report demonstrated that Th2-type inflammation promoted rapidly *in situ* proliferation of macrophages to avert potential tissue damage caused by universal recruitment of circulating inflammatory cells (25). In addition, extramedullary sites, such as the spleen, can generate bone marrow-derived monocytes and store, expand, and distribute in response to inflammatory signals (26, 27).

### Polarization of macrophages

Under pathological circumstances, TAMs played an indispensable role in the initiation and progression of lung tumors (28, 29). Since the discovery in the 1990s that IL-4 induces macrophage gene expression differently from classical gamma-interferon and bacterial lipopolysaccharide activation, this IL-4-inducible macrophage gene has been termed “alternative activated” macrophages (30). Meanwhile, macrophages are phenotypically and functionally heterogeneous, and macrophages can also be divided into two groups based on their phenotypic profile and local microenvironment: the pro-inflammatory “classically activated macrophages (CAM)” and the anti-inflammatory “alternately activated macrophages (AAM)” (31). CAMs perform the functions of immune surveillance and antigen presentation, secrete pro-inflammatory cytokines and chemokines, participate in positive immune responses. On the contrary, AAMs have a much weaker antigen-presenting ability, while playing an important role in immune regulation by secreting inhibitory cytokines such as IL-10 and/or TGF-β, downregulating anti-tumoral immune response. For surface biomarkers, CD14 is a common biomarker of monocyte/macrophages (32, 33), but the two subtypes of macrophages have differentiated expression of CD206, IL-10, and IL-12 (34, 35). CAM-type macrophages express MHC II, CD86, NO, iNOS, showing the characteristics of pro-inflammatory response and anti-tumor, while AAM-type macrophages express IL-10, arg-1, CD206, CD163, TGF-β, showing immunosuppressive and tumor-promoting characteristics (36). The classification of CAM-type macrophages and AAM-type macrophages was originally proposed for tissue macrophages and can also be extended to peripheral circulating blood monocytes (37). In the field of oncology, two macrophage subclusters were investigated, and the polarization of CAM towards AAM was reported to be correlated with poor prognosis and treatment failure (34, 36). Cellular and molecular mechanisms were reported. The cancer-AAM interactions facilitated the invasiveness of cancer cells and destruction of TME matrix in co-culture system (36). AAM also communicated with cancer cells by chemokines. Interleukin-6 secreted by AAM activated STAT3 signaling pathway and promoted proliferation and sphere formation of lung cancer cells (37).

### Mutual regulation between TAM and TME

Chronic inflammation and wound healing have a close relationship with carcinogenesis and tumor progression (38). TAMs are the most abundant immune cells in the TME, and have the characteristic of polarizing towards AAM-type macrophages. As a major component of infiltrating immune cells present in tumor tissue, TAMs are closely related to the inflammatory response in the tumor tissue, and aids tumor progression as well as metastasis (15, 16, 38). After being “educated” into TAMs, macrophages nourish the survival of tumor cells through various signaling pathways (15).

**Figure 1:**
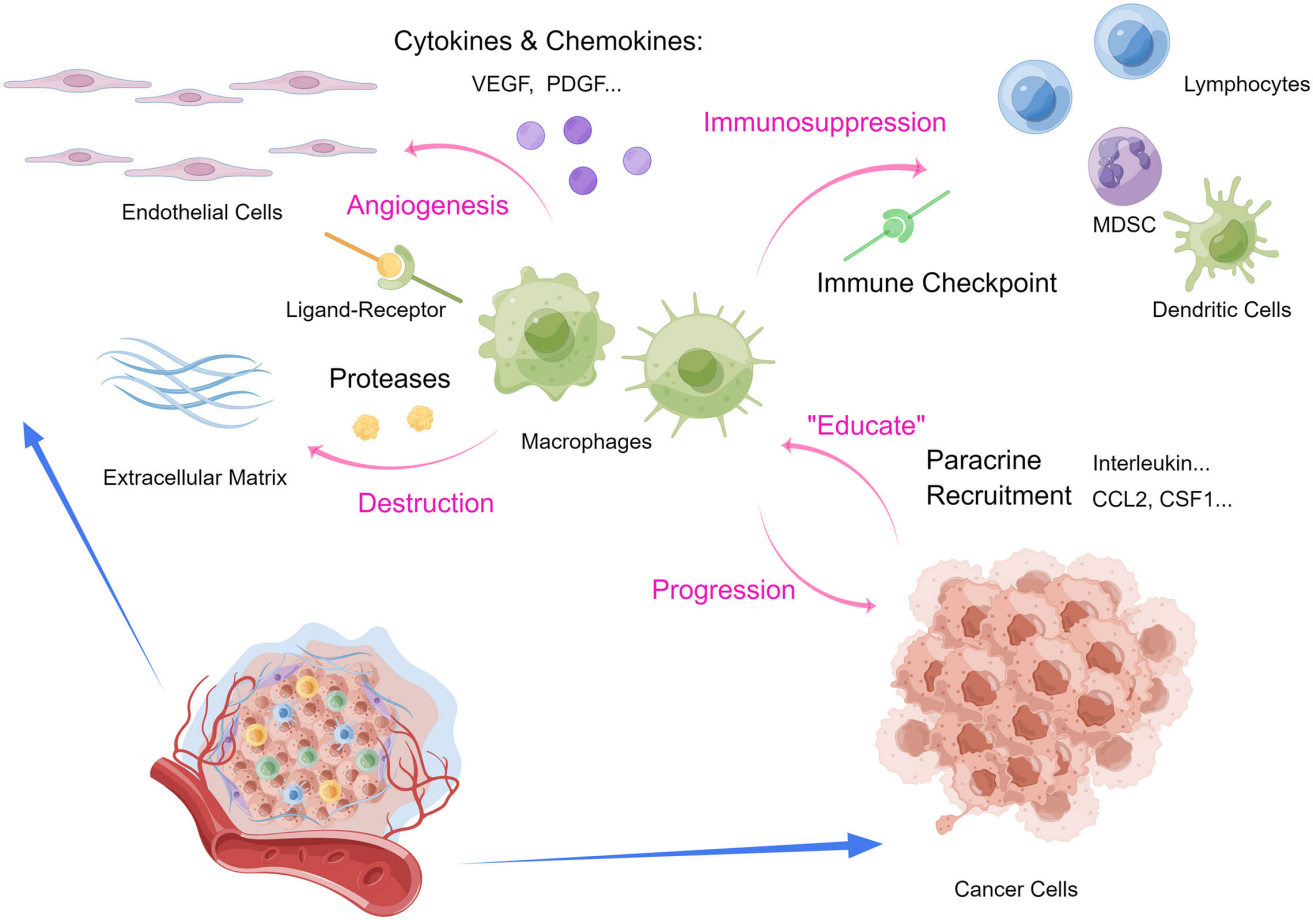
Interactions between macrophages and tumor microenvironment. (MDSC, myeloid-derived suppressor cells.) (By Figdraw.).

TAMs and tumor cells mutually promote each other through paracrine EGF/CSF-1 signaling (39, 40). Cancer cells secret CCL2 and CSF1 to recruit macrophages from circulating monocytes, and simultaneously IL-10 and PGE2 to facilitate immune evasion (41–43). To fuel tumorigenesis in the TME, TAM can secrete pro-angiogenic cytokines in the hypoxic TME including VEGFA, VEGFC and PDGF to facilitate tumor angiogenesis (14, 40). To destruct the tumor stroma, TAM also secrets proteases such as cysteine cathepsin and further promotes the invasion of cancer cells into the neo-vascularization to drive tumor progression (41). In addition to expressing VEGF-A and other angiogenic factors, TAMs also express Tie2 receptors that interact with endothelial cells and pericytes lining the tumoral vascularization to up-regulate angiogenesis (44). TAMs functions as a pivotal cellular component, in that macrophages also interact with other immune cells in the immunosuppressive TME. The PD-L1/PD-1 pair exists between the antigen-presenting TAMs and cytotoxic T cells, thereby inhibiting the antitumor effect of effector T cells (45, 46). Increased numbers of neutrophils are closely associated with poor prognosis in non-small cell lung cancer (NSCLC), possibly due to their expression of elastase that degrades the stroma in the microenvironment (47, 48). As tumors grow, immunosuppressive cells such as myeloid-derived suppressor cells (MDSCs) and regulatory T (Treg) cells enter the circulation in response to activate cytokine axes such as TGF-β and CXCL5-CXCR2 pathways (49). MDSCs and Treg cells infiltrate into the growing tumors, promote tumor angiogenesis and interferes with innate immunity by immune surveillance and antigen presentation, adaptive immunity *via* disrupting lymphocyte proliferation and biological functions, and damaging cytotoxicity of effector cells (50–53). Moreover, accumulated MDSCs can increase the degradation of stroma, thereby attenuating structural resistance for tumor proliferation, metastasis and angiogenesis (54, 55). In conclusion, TAM is the key to the immunosuppressive TME, and the crosstalk between TAM and various immune cells and TME cytokines plays an irreplaceable role. Understanding the main mechanisms by which TAMs are involved in tumor immunosuppression will help us improve clinical considerations and develop potential new strategies to overcome macrophage-related immune tolerance (**Figure 1**).

## Molecular mechanisms of AAM-type macrophage polarization

### IL-4, IL-13 signaling promotes polarization towards AAM macrophages

AAM-type macrophages involved in Th2-type polarization can help the body eliminate parasites, suppress inflammation, promote tissue repair, promote tumor growth, and participate in other immune regulations. Compared with the activation of CAM-type macrophages, the activation of AAM-type macrophages is relatively diverse. The polarization of AAM-type macrophages was first reported to result from the action of Th2-type cytokines IL-4 and IL-13 (30, 56, 57). The main receptors of the IL-4 signaling pathway are type I IL-4 receptors (IL-4Rα or IL-4Rγc) or type II IL-4 receptors (IL-4Rα or IL-13Rα1), while IL-13 signals through type II IL-4 receptor (58). The differential expression of type I or type II receptors on different cell types determines their different sensitivities to IL-4 and IL-13. Monocytes and macrophages have type I and type II receptors and are responsive to both cytokines (58, 59). IL-13Rα2, as a component of type II receptors, can act as a decoy for IL-13 and inhibit the selective activation of monocytes (60). The downstream signaling pathway of the IL-4 receptor involves the activation of multiple Janus kinases (56, 57, 61). Stat3 and Stat6 play crucial roles in AAM-type macrophage polarization (56, 62). Phosphorylated Stat is further transferred into the nucleus to regulate targeted genes involved in macrophage polarization (62). IL-10 secreted by Treg cells and B cells acts on IL-10R of macrophages, and then regulates Stat3 to promote the polarization of AAM-type macrophages and play an immunosuppressive role. In recent years, it has been found that Stat3 is highly activated in various tumor tissues (63, 64).

At the same time, other regulatory genes PPAR-γ, IRF4, JMJD3, and p50 are also involved in regulating the expression of AAM macrophage marker genes such as YM1, FIZZ1, Arg1, CCL17, and CCL22. STAT6 also induces the expression of the transcription factor PPAR-γ, which cooperates with STAT6 to regulate macrophage polarization and increase the expression of AAM-type biomarkers in a murine model of obesity (65). At the epigenetic level, the histone demethylase JMJD3 regulates the AAM macrophage-related genes Arg1, Chi3l3 (Ym1) and Retnla through the mutual change of histone H3 Lys4 (H3K4) and histone H3 Lys27 (H3K27) (Fizz1) transcription (66). IL-4 induces upregulation of JMJD3, which in turn reduces the histone methylation and activates transcription on the promoters of polarization driver genes (30, 62). IL-4 also causes activation of the PI3K signaling pathway, and studies have found that the PI3K subunit PI3Kγ promotes the polarization of AAM-type macrophages in pancreatic ductal carcinoma to exacerbate cancer progression (67, 68). The mutual regulatory function of Stat6 and PI3K in the induction of TAMs polarization has not been directly reported, but IL-4 has been shown to be an important mediator of TAMs polarization in some murine tumor models (59).

Notably, molecular interactions of various signaling pathways also promoted the AAM polarization in the TME. For example, studies found that IL-4 induce the IRF4 expression to promote macrophage polarization not only by Stat6 or PI3K signaling pathway, but also by metabolic regulation such as glycolysis (69), and IRF4 has been reported to be a contributing factor of AAM-type polarization (70). To sum, IL-4 and IL-13 mediated macrophage polarization toward the anti-inflammatory and pro-tumoral phenotype, and function as pivotal molecules connecting several mechanisms.

### Other elements inducing AAM polarization

According to different activation mechanisms, AAM macrophages can be further divided into three subtypes: M2a, M2b, and M2c (71). M2a macrophages are mainly stimulated by Th2 cytokines represented by IL-4 and IL-14 (72). M2b macrophages are induced by ICs and agonists of Toll-like receptors (TLRs) or IL-1R (15, 71). M2c macrophages are activated by IL-10, transforming growth factor-β (TGF-β), and glucocorticoids (GCs) to antagonize effector cells and induce immune regulation (71, 73).

Immune complexes can promote the polarization of AAM macrophages through FcγR. Binding of immune complexes to activated FcγRs on macrophages triggers a tyrosine kinase Syk-dependent pathway that not only inhibits TLR4 signaling but also inhibits type I interferon through upregulation of IL-10 and negative regulation of A20, ABIN3 and SOCS3 type interferon signal, indicating an increased biological effect of anti-tumoral macrophages (74). Ligation of immune complexes to the inhibitory receptor FcγRIIb on macrophages induces prostaglandin E2 production, thereby inhibiting TLR4-triggered expression of inflammatory cytokines such as IL-6 and TNF7 (75).

Reprogramming metabolism is an emerging hallmark of cancer (76). Cancer cells alter their metabolism to adapt to their microenvironment and facilitate immune evasion. Tumor-derived metabolic factors play key roles in regulating macrophage polarization (77). For instance, lactic acid is highly enriched in the TME due to the intense energy production by glycolysis (78). Lactic acid derived from malignant tumor tissues is found to promote tumor progression by promoting macrophages polarization (79). In addition, lactic acid was shown to drive TAM proliferation during EMT (80). These studies collectively demonstrate a role of lactic acid and glucose metabolic reprogramming in macrophage polarization.

**Figure 2:**
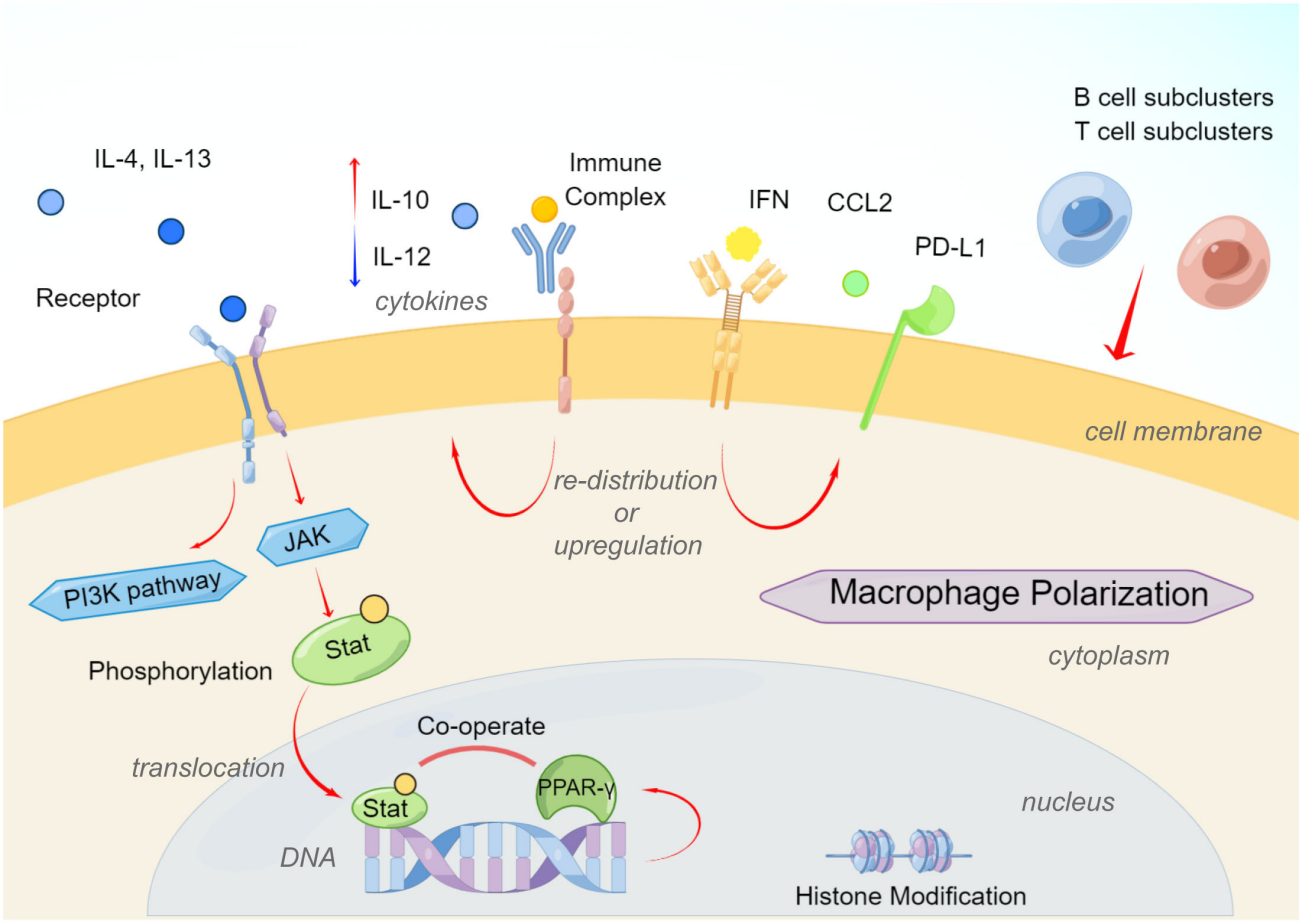
Brief mechanisms of macrophage polarization from CAM to AAM. (By Figdraw.).

Research on tuberculosis reported that B cells also take part in modulating the phenotype and functions of macrophages (81). In the inflammation milieu, B cells produced type I IFN *via* STING pathway, triggered the preference for M2 polarization and activated the regulatory macrophages (81). In addition, Treg cells also significantly affect the function of macrophages. Human monocytes co-cultured with CD4+CD25+Foxp3+ Treg cells presented high expression of M2 biomarkers (such as CD163, CD206 and CCL18), low expression of inflammatory cytokines such as TNF, IL-1β, IL-6 and CCL3, and were more prone to polarize into AAM-type macrophages (82). Treg cell-driven IL-10 is involved in the suppression of inflammatory cytokines and the expression of CD163 and CCL18 (82, 83). CD4+CD25+ T cells were found to polarize tissue macrophages into AAM-type through arginase, IL-10 and TGF-β pathways (84). In contrast, AAM-type macrophage polarization not only drives the differentiation of CD25+GITR+Foxp3+ Treg cells, but also regulates their recruitment by releasing CCL22 (64, 85). Moreover, research demonstrated that HIV infection up-regulated PD-1 ligation and promoted the recruitment of IL-10-releasing monocytes, and these two molecules synergized to potentiate AAM polarization *via* ligand-receptor pair and in the milieu (86) (**Figure 2**).

## Therapeutic strategies targeting AAM-type macrophage polarization

As mentioned above, TAMs account for an important proportion of the entire tumor microenvironment, and they are involved in various aspects of tumor progression. Immunotherapy targeting TAMs is gradually becoming a research hotspot. Herein, we discuss potential therapeutic strategies targeting AAM-type macrophage polarization in lung cancer.

### CCL2 monoclonal antibody or CSF1R inhibitor

Since chemotaxis is the main contributing factor driving monocyte recruitment and colonization, chemokines regulating chemotaxis become targets to inhibit the subsequent phenotypes and functions of macrophages. The monoclonal antibody CNT0888 (carlumab) targeting CCL2 has been investigated in clinical trials and showed good efficacy and tolerability in patients with advanced malignant tumors (87). Inhibition to CSF1R pathway also attenuated macrophage polarization. There are two ways to inhibit the CSF1/CSF1R signaling pathway: direct inhibition to CSF1R tyrosine kinase, indirect blocking CSF1 from binding to CSF1R. Many inhibitors of the CSF1/CSF1R signaling pathway have been reported, most of which are small-molecule heterocyclic compounds with different scaffold structures. The phosphorylation process of tyrosine residues can achieve the effect of receptor inactivation (88). CSF-1R tyrosine kinase inhibitors that block the CSF-1 signaling pathway have shown good therapeutic effects in preclinical models of various tumors, including acute myeloid leukemia, malignant melanoma, and malignant glioma (88, 89), and CSF-1R inhibitor RG7155 significantly reduced the macrophage infiltration in a case of sarcoma with high CSF-1 expression (90). AZ683 is a potent and highly selective CSF1R inhibitor with good oral bioavailability. *In vivo* experiments show that AZ683 can effectively inhibit TAMs and exert anti-tumor effect (91). However, the latest data show that the therapeutic effect of these antibodies and inhibitors is not durable, and it is easy to relapse and aggravate the disease after treatment is completed. In lung cancer treatment, preclinical study has suggested that CSF1R inhibition by BLZ945, a CSF1R inhibitor, substantially limits malignant pleural effusion formation induced by lung adenocarcinoma (92). PLX647 is a pyrrolopyridine compound that can bind to the juxtamembrane domain of the kinase to maintain the autoinhibitory state of the protein, thereby inhibiting the phosphorylation of CSF1R with an IC50 of 28 nmol/L. PLX647 improves systematic immunosuppressive state by inhibiting CSF1/CSF1R signaling and has been shown to be effective in the treatment of breast cancer, melanoma and lung cancer (88). The presence of TAMs will also affect the efficacy of chemotherapy drugs. Studies have confirmed that during treatment of docetaxel, CSF-1 monoclonal antibody or CSF-1R blockade will improve the anti-tumoral effects of paclitaxel, and since TAMs secrete the immunosuppressive molecule IL-10, thereby blocking IL-10 combined with docetaxel resulted in better clinical outcomes (93). Therefore, reducing the infiltration of TAMs directly or indirectly will improve the therapeutic effect of malignant tumors.

### Re-acclimation of AAM-type macrophages and new strategies

Besides inhibiting TAM infiltration, alternatively, re-educating TAMs by immune checkpoint inhibitors or TAM surface biomarkers reactivate the antitumoral activity of TAMs and relieves their immunosuppressive function. The immune checkpoints on TAMs that have been discovered so far include PDL1, CSF1R, Dectin-1, PI3Kγ, etc., and the corresponding inhibitors and antibodies have achieved good therapeutic effects in clinical practice (94, 95). In the field of immunotherapy, current strategies mainly include blocking immune checkpoints such as PD-1 and CTLA-4 by antibodies or small-molecule inhibitors, thereby “re-firing up” the anti-tumor immune response. PD-1 is mainly expressed in activated T cells and is an important immune checkpoint receptor. After PD-1 binds to its ligands PD-L1 or PD-L2 in tumor cells and tumor microenvironment, it transmits inhibitory signals to T effector cells, hinders T cell survival, and facilitates immune tolerance (96, 97). Chronic exposure to inflammatory cytokines and high levels of antigens can also lead to increased expression of PD-1 and PD-L1, which are hallmarks of T cell exhaustion and dysfunction (98). It was found that PD-1 blocks proximal activation of PI3K/Akt signaling pathway, and the extent of T cell inhibition depends on the signaling of T cell receptors (99). Immune checkpoint antibodies currently in development or clinically approved include the PD-1 antibodies nivolumab and pembrolizumab and the PD-L1 antibodies atezolizumab, durvalumab, and avelumab (100). The latest research has found that combining these immune checkpoint inhibitors and antibodies with chemotherapy or targeted therapy shows synergistic effects.

In recent years, with the rapid development of tumor immunity research, immune checkpoint inhibitors such as CTLA-4 antibody and PD-1/PD-L1 antibody have been successfully applied in a variety of cancers, such as melanoma, non-small cell lung cancer, advanced cervical cancer, hepatocellular carcinoma, skin squamous cell carcinoma, bladder cancer, etc. Immunotherapy has become one of the main treatment options for patients with advanced cancer. Among them, the combination therapy of immune checkpoint inhibitors with precision and multi-pathway targeting has unique advantages in overcoming drug resistance and enhancing the specific recognition and killing of tumor cells by immune cells (101). For example, the combination of nivolumab, a PD-1 inhibitor, and ipilimumab, a CTLA-4 inhibitor, can prolong the progression-free survival of lung cancer patients with good complementarity. Nivolumab combined with LAG-3 inhibitor BMS-986016 in the treatment of advanced melanoma can effectively overcome the resistance of PD-1 monotherapy. The combination of PD-1 inhibitor and TIM-3 inhibitor in the treatment of non-small cell lung cancer can inhibit the resistance to PD-1 inhibitor (102). The combination of CTLA-4 inhibitor and LAG-3 inhibitor can induce immune tolerance through co-inhibiting signaling pathway. The combination with IDO inhibitor can effectively reduce the tumor volume and prolong the survival time of a melanoma murine model. In addition, the emergence of bifunctional antibodies with good targeting property, which can effectively exert synergistic effects through dual-pathway or dual-target blocking, has given new enlightenment to cancer treatment, and may become one of the key therapeutic strategies for human to conquer cancer.

The existing treatment strategies have their own advantages and disadvantages. In order to better improve the tumor treatment effect, new treatment strategies are future-oriented. For example, to improve the “phagocytic ability”, in a physiological state, normal cells have a “phagocytic checkpoint”, that is, the expression of anti-phagocytic molecules to avoid the self-elimination of phagocytic cells, and tumor cells also rely on this phagocytic checkpoint to carry out immune evasion. Therefore, the identification and intervention of phagocytic checkpoints may provide a new method to re-educate TAMs to restore the phagocytosis against tumor cells. For example, under immunosuppressive conditions, the cancer cell membrane protein CD47 can recognize SIRPα on the surface of macrophages to form the CD47-SIRPα signaling complex, inhibiting the phagocytosis of tumor cells by macrophages and enabling tumor cells to escape immune surveillance for tumor development (103, 104). Therefore, CD47-SIRPα blocking antibody may restore the phagocytosis of macrophages. Furthermore, given that TAMs have the ability to phagocytose nanoparticles, nanoparticles are ideal therapeutic targets. Nanoparticles containing tumor peptides are used to promote the recording of TAMs, and the characteristics of nanoparticles targeting TAMs can be used to promote antitumor immunity.

## Biological features of Tim-3 positive TAMs

In recent years, Tim-3 positive macrophages have attracted great attention. The discovery of immune checkpoint molecules and the elucidation of their functions have provided new targets and therapeutic methods for tumor therapy, such as CTLA-4, PD-1, Lag-3 and Tim-3. Tim-3 belongs to the immunoglobulin superfamily (IgSF), which consists of four known domains, including a variable immunoglobulin domain (IgV), a mucin domain, a transmembrane domain, and a cellular inner tail region (105). In the immune system, Tim-3 was initially identified as a specific membrane marker selectively expressed on IFN-γ-producing CD4+ helper T cells (Th1) and CD8+ cytotoxic T cells (Tc1) (105). Later research on tumor microenvironment demonstrated that Tim-3 is expressed by other cell types, such as natural killer cells (NK cells), dendritic cells (DC cells), monocytes, macrophages, and even different types of tumor cells (106, 107). The study of Anderson et al. showed that Tim-3 can be highly expressed on macrophages and promote the inflammatory response of macrophages through the NF-κB pathway (108). Tim-3 expression can be used as an independent prognostic factor in colon cancer patients, and Tim-3 can directly promote tumor growth through STAT3 or STAT3-pSTAT3 pathway. Researchers detected the expression of Tim-3 in tumor-associated macrophages in lung cancer tissues, and in CD68+ tumor-associated macrophages, lung cancer patients with high Tim-3 expression had shorter OS and poorer prognosis (109). The specific mechanism of Tim-3-positive macrophages in lung cancer is still unclear, but its findings in other tumors can provide ideas for our follow-up research. Tim-3 expression on TAMs in hepatocellular carcinoma is induced by tumor-derived signals including TGF-β (110). This further promotes TAM-mediated growth of HCC due to the secretion of soluble factors such as IL-6. Some studies have found that TLR ligand lipopolysaccharide can inhibit the expression of TIM-3 protein in macrophages and restore the immune activity of macrophages (107). This suggests that the expression of TIM-3 may be related to the TLR expression and its downstream signaling pathways. In addition, in HCC, TIM-3 protein regulates the transformation of CAM macrophages towards AAM macrophages, which further inhibits the inflammatory response (110, 111). Secondly, TGF-β-mediated Tim-3 expression in turn regulates the ability of macrophages to secrete cytokines *via* the NF-κB-IL-6 pathway. Researchers detected Tim-3 expression on tumor cells and CD204+ tumor-associated macrophages in clear cell renal cell carcinoma, and found that higher Tim-3 expression levels were associated with shorter PFS in patients, and similar to reports on lung cancer, Tim-3 was found to induce resistance in renal cancer cells to standard treatments as sunitinib and mTOR inhibitors (112). Based on previous findings, we hypothesized that Tim-3 may directly promote tumor growth through the IL-6-STAT3 pathway or the NF-κB-IL-6 pathway, or negatively regulate anti-tumor immunity, thereby facilitating tumor immune escape and promoting tumor cell growth.

In addition, 1 ug/ml LPS treated macrophages for 6 h not only up-regulated TLR4 and MyD88 mRNA expressions, but also significantly up-regulated Tim-3 mRNA expression, indicating that activation of TLR4 signaling pathway can regulate the expression of Tim-3 on the surface of macrophages. Yang et al. used LPS to treat peritoneal macrophages derived from a mouse model of sepsis for 4 hours and found that the expression of Tim-3 mRNA on the cell surface was significantly up-regulated, but they used the same concentration of LPS to treat mouse-derived RAW264.7 cells and found that Tim-3 mRNA expression was down-regulated with the increase of LPS concentration, and decreased to the lowest level at 100ng/ml, suggesting that the regulation of Tim-3 by TLR4/LPS signaling pathway is closely related to the cell origin, and this signaling pathway affects macrophages from different sources (113).

Besides its role in tumor progression, macrophages are also involved in other pathological conditions. Monney et al. established an experimental mouse model of autoimmune encephalomyelitis and showed that Tim-3 can promote the massive activation and proliferation of monocyte-macrophages and promote the inflammatory response (106). The Tim-3-galectin-9 interaction can also transduce reverse signaling, and a murine model of pulmonary infection with Mycobacterium tuberculosis (Mtb) has also shown that the Tim-3 signaling pathway can activate macrophages and activate innate immune responses (114). Tim-3 is essential for the induction of IL-1β and enhanced macrophage anti-mycobacterial activity through a galectin-9-dependent mechanism. When mycobacterium tuberculosis-infected cells were treated with the Tim-3 fusion protein. In the case of macrophages, fewer CFUs were recovered in this case. Tim-3 is essential for induction of IL-1β and enhanced macrophage anti-mycobacterial activity through a galectin 9-dependent mechanism. Zhang et al. showed that blocking or silencing Tim-3 on the surface of macrophages can induce increased secretion of pro-inflammatory factors IL-12 and IL-6, as well as increased secretion of anti-inflammatory factor IL-10. The authors speculate that the regulatory role of Tim-3 on immune inflammation is influenced not only by Tim-3 expression itself, but also by the state of macrophages and the balance between inhibitory and stimulatory molecules involved (115). Tim-3 expression is lower on M1 macrophages that have multiple functions (eg, phagocytosis, antigen presentation, and production of pro-inflammatory cytokines) and are used to eliminate cancer cells. To illustrate the immunosuppressive role of Tim-3 in various cell types and its role in regulating immune cell cross-talk in the tumor environment.

Extensive preclinical data support that blocking the TIM-3 signaling pathway may promote immune cells to mediate anti-tumor responses, and can be combined with other immune checkpoint receptor blockers to further enhance the anti-tumor effect. Preliminary signs of clinical efficacy have also been observed in patients with solid tumors, including NSCLC, who received sabatolimab and anti-PD-1 antibody spartalizumab, suggesting that blockade of TIM-3 might represent a potential therapeutic strategy in lung cancer (116).

## Summary

TAMs are important immune cells in the immune microenvironment of lung cancer with high heterogeneity. The polarization of macrophages and related mechanisms play an important role in the progression of lung cancer. In this review, we aimed to overview the current understanding of TAMs in the context of lung cancer. First, we discussed mutual regulation between TAM and TME, and established the key role of TAM and TME in supporting tumor cell survival: TAM nourishes tumor cell survival through a large number of signals from the TME, and in turn regulate the TME from many aspects. We also described the molecular mechanism of AAM polarization and therapeutic strategies for cancer-promoting AAM macrophages, including CCL2 monoclonal antibodies or CSF1R inhibitors, AAM re-acclimation targeting immune checkpoints, and new strategies to improve the “phagocytic ability” of cells. Finally, we discussed the involvement of TIM-3 positive macrophages in cancer pathogenesis, and explored TIM-3 inhibition as a potential therapeutic strategy for lung cancer. The extensive involvement of TAM in cancer pathogenesis and promising preclinical and early clinical data summarized above have emphasized the opportunities of the development of AAM-targeting therapeutic strategies against lung cancer.

## Author contributions

QX, QH, and RW provided the direction and guidance for this manuscript. QH and RW wrote the whole manuscript. ZZ and HM were responsible for the collation of the paper. GW made significant revisions to the manuscript. All authors contributed to the article and approved the submitted version.

## Funding

This work has been funded with support from the Research Center of Clinical Medicine of Affiliated Hospital of Nantong University, Nantong, China. The funders had no role in the study design, data acquisition, data interpretation, or writing of the manuscript.

## Conflict of interest

The authors declare that the research was conducted in the absence of any commercial or financial relationships that could be construed as a potential conflict of interest.

## Publisher’s note

All claims expressed in this article are solely those of the authors and do not necessarily represent those of their affiliated organizations, or those of the publisher, the editors and the reviewers. Any product that may be evaluated in this article, or claim that may be made by its manufacturer, is not guaranteed or endorsed by the publisher.

## Abbreviations

EMT, Epithelial-to-mesenchymal transition; TME, Tumor microenvironment; TIM-3, T cell immunoglobulin and mucin domain-3; SCLC, small cell lung cancer; NSCLC, non-small cell lung cancer; LUSC, lung squamous cell carcinoma; ECM, extracellular matrix; NK cells, natural killer cells; DC cells, dendritic cells; MDSC, myeloid-derived suppresser cells regulatory T Treg cells; TAM, tumor-associated macrophages; MPS, mononuclear phagocytic system; CAM, classically activated macrophages; AAM, alternately activated macrophages; MDSCs, myeloid-derived suppressor cells; TLRs, Toll-like receptors; TGF-β, transforming growth factor-β; GCs, glucocorticoids; IgSF, immunoglobulin superfamily; IgV, immunoglobulin domain; CVB3, coxsackievirus B3; Mtb, mycobacterium tuberculosis; MDSC, myeloid-derived suppressor cells.
